# Continuous CF/PA6 Composite Aircraft Window Frame Manufactured via a Novel Winding Compression Process

**DOI:** 10.3390/ma17061236

**Published:** 2024-03-07

**Authors:** Hongfu Li, Zengru Ge, Yanfei Zhang, Boming Zhang, Ying Wu

**Affiliations:** 1 School of Materials Science and Engineering, University of Science and Technology Beijing, Beijing 100083, China; 2 COMAC Beijing Aircraft Technology Research Institute, Beijing 102211, China; gezengru@comac.cc; 3 School of Materials Science and Engineering, North University of China, Taiyuan 030051, China; zhangyanfei208@sina.com; 4 School of Materials Science and Engineering, Beihang University, Beijing 100083, China; zbm@buaa.edu.cn

**Keywords:** thermoplastic composites, aircraft window frame, winding compression molding, mechanical property, finite element

## Abstract

Using fiber-reinforced polymer composite to replace metal in window frames has become a trend in aircraft manufacturing to achieve structural weight reduction. This study proposes an innovative winding compression molding process for continuous production of aircraft window frames using continuous carbon fiber-reinforced polyamide 6 thermoplastic composite filaments (CF/PA6). Through process parameter optimization, the production cycle of CF/PA6 composite window frames was controlled within 5 min, with an ultra-low porosity of 0.69%, meeting aviation application standards. Combining mechanical property experimental tests and finite element analysis, the mechanical performance of window frames made from three different materials was compared and evaluated. In the hoop direction, the mechanical performance of the continuous CF/PA6 thermoplastic window frames were significantly higher than that of chopped CF/epoxy compression molding window frames and aluminum alloy window frames. In the radial direction, the maximum strain occurred at the corner with the highest curvature of the frame due to the absence of fiber reinforcement, resulting in weak pure interlayer shear. Nevertheless, the thermoplastic CF/PA6 winding compression molded window frame still exhibited a high resistance to crack propagation and damage, as evidenced by the absence of any detectable sound of microdamage during testing with a 9000 N load. It is believed that achieving a further-balanced design of hoop–radial performance by appropriately introducing radial ply reinforcement can lead to a significant weight reduction goal in the window frame. The findings in this study provide an innovative process reference that can be universally applicable to high-speed and near-net-shape manufacturing without material waste of continuous fiber-reinforced thermoplastic composite products.

## 1. Introduction

Lightweight design is a crucial approach to improving the thrust–weight ratio and fuel economy in the aerospace field. The commonly used materials in lightweight design include Al alloys, Ti alloys, high strength steel, and composites [1]. Among them, carbon fiber-reinforced polymer composites (CFRP) possess low density, no more than 1.8 g/cm^3^, but with high strength, high damage tolerance, improved fatigue resistance, corrosion resistance, and moisture resistance, making them indispensable materials for weight reduction in the aviation field [2]. For instance, the usage of fiber composites in aircraft like the Airbus A350 and Boeing 787 has surpassed 50 wt %, including structural components of the wing box, empennage, and fuselage, as well as the control surfaces, setting a benchmark in the commercial aircraft industry.

Aircraft windows, as crucial components of the fuselage, serving the dual functions of structural support and human–machine interaction, are also a key aspect of weight reduction for commercial aircraft [1,3]. Presently, civilian aircraft models employing composite material window frames include the Boeing 787 [4] and Airbus A350 [5]. Considering the balance of production efficiency, cost, and maturity of composite processing technology, the 787 Dreamliner utilized chopped CF/epoxy composite compression molding technology in its window frames. Compared to traditional aluminum alloy frames, the novel composite aircraft window frames reduce weight by 50% and offer higher damage tolerance, providing excellent fuel economy. Meanwhile, they enhance the flight experience for passengers by providing a larger view of the scenery outside [6,7]. However, there are two aspects that are worth improving. Firstly, compared to continuous fiber reinforcement, the strength and modulus levels of short fiber composites are still relatively low, thus failing to fully exploit the maximum weight reduction potential of composites. Secondly, the thermosetting composites used have long curing times and scrap rates, and are not recyclable after retirement. As a comparison, the route employed in the manufacturing of Airbus A350 window frames provides a new technical reference by utilizing automatic continuous carbon fiber layup-sewing followed by epoxy resin high-pressure resin transfer molding (HP-RTM). Airbus A350 window frames manufacturing provides a new technical reference by using automatic continuous carbon fiber layup-sewing with followed by epoxy resin HP-RTM infusion process. However, the layup-sewing efficiency is low and the method also faces the issue of how to recycle thermosetting composites.

In contrast, continuous fiber-reinforced thermoplastic composite materials (CFRTP) can effectively address the aforementioned mechanical property, manufacturing efficiency, and recycling concerns [8]. The specific strength and specific stiffness are several times higher than those of short fiber reinforcement. Additionally, CFRTP is expected to achieve much faster forming compared to thermosetting composites due to the physical changes in thermoplastic polymers during molding, without the need for curing [9,10,11]. Additionally, the intrinsic recyclability of thermoplastic composites aligns with the concept of green and low-carbon development globally. More and more fields are in favor of developing and using thermoplastic composite materials, structures, and manufacturing processes, aiming to achieve weight and cost reduction and to minimize the environmental impact through eco-design and energy consumption optimization all along the life-cycle (towards zero impact) [12,13,14,15,16]. It is also recognized as the mainstream composite material system for the next generation of aircraft. Typical examples are the programs of TAPAS and Clean Sky, led respectively by Boeing and Airbus, which have been conducting foundational applied research and validation for nearly two decades. To address the aforementioned issues of window frames, a sub-project named WINFRAME 4.0 was developed to fabricate a kind of carbon fiber-reinforced thermoplastic polyphenylene sulfide (CF/PPS) composite window frame for the Green Regional Aircraft demonstrator in Clean Sky [17,18]. The CF/PPS composite window frames were manufactured following an innovative molding process named the Quilted Stratum Process (QSP) [19]. It can produce one window frame every 10–20 min, achieving a much shorter manufacturing cycle time of parts than currently used processes to manufacture composite window frames such as RTM that the A350 employs. However, there is still room for improvement in the production cycle. More importantly, the use of prepreg with fabric forms results in significant waste during circular cutting. Therefore, the development of a new type of thermoplastic composite window frame molding technology, which integrates performance, efficiency, net-shaping, and low-carbon friendliness, remains to be explored.

Currently, the manufacturing of circumferential or rotary structures resembling window frames with CFRTP is typically achieved through winding or automated laying processing technologies [20,21,22]. However, to achieve high interlayer quality, auxiliary equipment such as flame, laser heating, and rollers are often needed, leading to lower molding efficiency [23]. Alternatively, a preformed composite semi-product can be first created using automated fiber placement (AFP) and then undergo a secondary hot-stamping molding. However, expensive equipment investment is usually required for AFP [24]. Hence, in this paper, we propose an innovative and low-cost thermoplastic composite window frame-forming process that approaches net shaping through a combination of winding and compression molding processing: continuous carbon fiber-reinforced polyamide 6 (CF/PA6) thermoplastic composite filaments were prepared in advance, then a rough window frame preform was prepared through a winding process, and then the thermoplastic window frame was finally obtained by further using a rapid hot-in and cold-out compression molding technology. Simulation and performance comparison of window frames manufactured from winding compression CF/PA6, chopped CF/epoxy sheet molding, and aluminum alloy were conducted. Preliminary experimental results demonstrate that the entire molding cycle of winding compression processing can be controlled within 5 min, which is competitive to CF/epoxy sheet molding, alongside achieving high molding quality and with low equipment investment. Our findings provide a novel reference for the forming of circumferential or rotary structures manufactured from continuous fiber thermoplastic composites with high-performance and rapid molding.

## 2. Materials and Methods

### 2.1. Preparation of the Window Frame

The winding compression technology proposed in this paper consists of three steps: filament impregnation, preform winding, and compression molding. Specifically, it first refers to the preparation of pre-impregnated continuous carbon fiber-reinforced thermoplastic composite filaments, such as CF/PA6 filament with 2 mm diameter, in this paper, followed by a rapid filament winding forming to prepare a preform with a rough apparent shape, such as aircraft window frame. Subsequently, this rough preform is reheated and placed in a high-precision final product molding cavity for neat compression shaping, further compacting the composites and improving the weak interface layer between winding layers or fiber bundles under high pressure. Consequently, products with high performance and low porosity are obtained. Thanks to the processing advantages of thermoplastic composites without a chemical curing process and the high fault-tolerance of the preforms without strict porosity requirements, efficient winding process parameters can be applied, achieving high producing efficiency. The three steps are respectively depicted as follows.

#### 2.1.1. Preparation of the CF/PA6-Impregnated Filaments

Continuous CF/PA6 pre-impregnated filaments with diameter of 2 mm were prepared using a melt impregnation method. Specifically, three bundles of 12 K carbon fibers (Toray T700SC-12K), i.e., 36 K in total, preheated to 150 °C, were continuously passed through cross-head impregnation dies at a speed of 5 m/min. In the dies, the fibers were thoroughly impregnated with molten PA6 (UBE 1013B, T_m_ = 220 °C) provided by an extruder. Fiber content control was achieved through a shaping die with a 2.0 mm aperture, followed by rapid cooling for the filament shaping. Finally, the continuous CF/PA6 filaments ready for preform winding were obtained through traction and winding into rolls.

#### 2.1.2. Winding Molding of the Window Frame Preform

**Figure 1:**
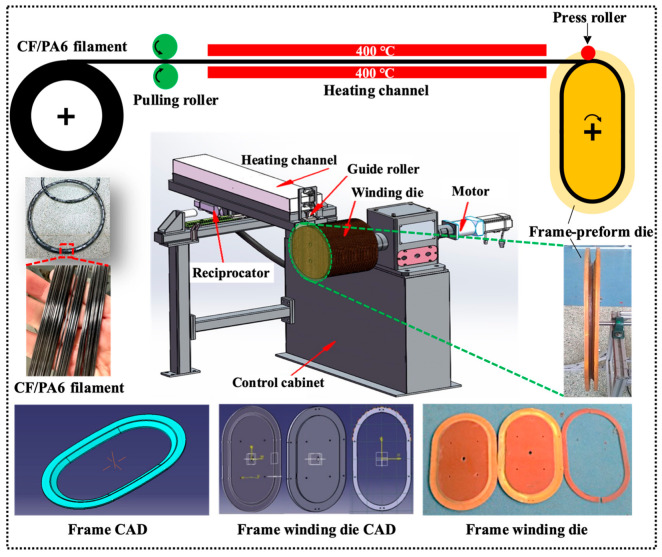
Winding process of the window frame preform manufactured with continuous CF/PA6 thermoplastic composite filaments.

The experimental production line for the continuous winding of continuous fiber thermoplastic filaments for window frame preforming is depicted in Figure 1. The equipment mainly consists of unwinding devices, traction rollers, a heating channel, guiding rollers, pressure rollers, winding molds, and winding machines. The specific procedural steps are as follows: Continuous CF/PA6-impregnated filaments, under the power of the traction roller and a pre-tension force of 13 N, continuously pass through a 400 °C infrared heating channel at a speed of 10 m/min to undergo thorough remelting. Subsequently, the bundles are positioned by guiding rollers onto the rotating winding mold core. With the combined action of radial rolling pressure rollers and a reciprocating mechanism, the preforming process is completed.

#### 2.1.3. Compression Molding of the Final Window Frame

**Figure 2:**
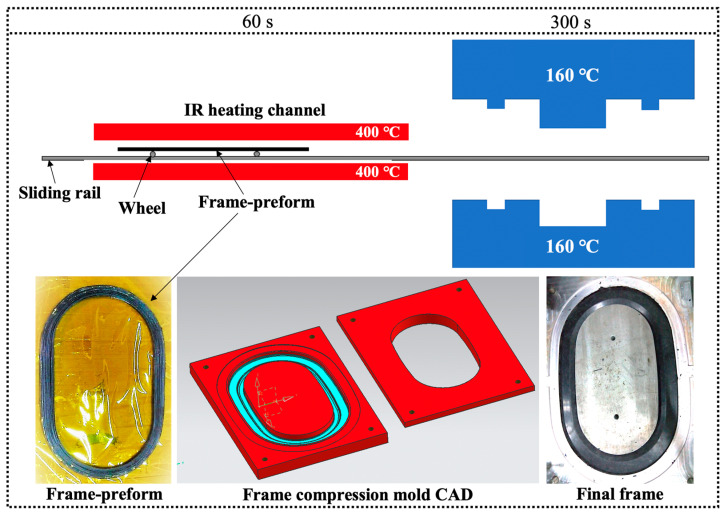
Rapid cold compression process of the final window frame through a wound CF/PA6 frame preform.

The final shaping of the window frames was performed using a rapid stamping process, fully exploiting the forming advantages of thermoplastic composite materials to ensure high forming efficiency. As seen in Figure 2, the compression molding equipment mainly consists of an infrared heating channel, preforming body clamping fixture, sliding guide rails, molds, and a compression molding machine. The specific procedural steps are as follows: Firstly, the preformed window frame, which has been wound into a rough shape, is installed on the clamping fixture of the sliding guide rail. After heating to 260 °C under the 400 °C infrared heater, the preformed window frame is quickly slid into the cavity of the compression molding tool at 160 °C. It is then kept under 4 MPa pressure until the temperature of the window frame drops to below 170 °C. Afterward, the continuous CF/PA6 thermoplastic composite window frame sample is removed and placed in a pressurized forming mold with a lower temperature of 80 °C. After cooling to 80 °C, it is taken out. This two-step molding–cooling method can effectively avoid large deformations induced by the crystallization shrinkage and cooling shrinkage of CF/PA6 consolidation from 170 °C to 80 °C. In addition, it can significantly reduce the mold occupancy time of window frame molds, thereby improving manufacturing efficiency and production cycle. To facilitate easy component demolding, two plies of polyimide (Kapton, DuPont, WA, USA) and a release agent (FREKOTE, Henkel, Düsseldorf, Germany) were placed in both the male and female tooling parts in advance. To determine the compression molding process parameters, the influence of compression molding temperature and holding pressure time on the surface forming quality and mechanical properties of the material were simultaneously investigated.

#### 2.1.4. Preparation of Chopped CF/Epoxy Composite Frame

For comparison, short carbon fiber-reinforced epoxy composite material (CF/epoxy) window frames were also prepared and tested. Based on the Lytex Molding Guidelines provided by Quantum Composites for Lytex^®^ 4149 (Bay, MI, USA) the material preheating temperature was determined to be 90 °C for 2 min, and the feeding weight was calculated as *V*_sample_ × density × 1.05%, where *V*_sample_ is the volume of the frame. The molding temperature was set at 150 °C, the molding pressure at 7 MPa, and the holding pressure time at 5 min, and the mold extrusion surface was vented.

### 2.2. Characterization

#### 2.2.1. Fiber Content

According to the experimental requirements in this paper, the fiber volume fraction of the composite material was calculated or tested using the following three methods.

(1) Density method

Firstly, the material density is determined using the Archimedes buoyancy method. Then, the volume fraction of the fibers in the composite material is calculated using Equation (1).

(1) $$V_{f}=\frac{\rho -\rho_{r}}{\rho_{f}-\rho_{r}}\times 100\%\;$$

where *ρ_f_* is the fiber density (1.80 g/cm^3^ for carbon fibers), and *ρ_r_* is the resin density (1.13 g/cm^3^ for PA6).

(2) Weight-loss method

**Figure 3:**
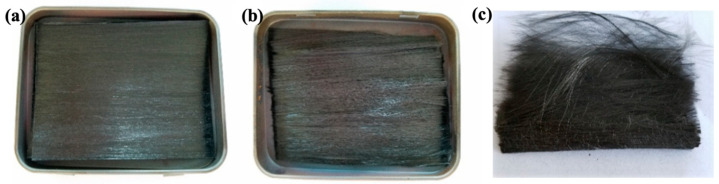
Muffle furnace weight-loss method used for measuring the fiber content of CF/PA6 composites: (**a**) the obtained CF/PA6 composite, (**b**) the residual after weight-loss, and (**c**) the residual CFs.

As shown in Figure 3, a certain mass of the composite material sample, denoted as *m*_1_, is weighed and then placed in a crucible with a mass denoted as *m*_2_. The crucible is heated in a Muffle furnace at 650 °C for 30 min. The remaining total mass of the crucible and the residue is recorded as *m*_3_. The mass fraction, *M_f_*, and volume fraction, *V_f_*, of carbon fibers in the CF/PA6 composite material are calculated using Equations (2) and (3).

(2) $$M_{f}=\frac{m_{3}-m_{2}}{m_{1}}\times 100\%\;$$

(3) $$V_{f}=\frac{M_{f}\rho_{r}}{{M_{f}\rho_{r}+M_{r}\rho }_{f}}\times 100\%\;$$

where *M_f_* is the fiber mass fraction, *M_r_* is the resin mass fraction, and *V_f_* is the fiber volume fraction. Since the burning process may involve the oxidation of carbon fibers, the fiber content calculated by this method may be slightly lower compared to the other two methods.

(3) Metallographic imaging method

Composites with specific dimensions are vertically embedded in epoxy resin. After the epoxy resin cures, the cross-section is polished, and optical micrographs are taken using a microscope (DM4000M, Leica, Germany). Subsequently, Image J software v1.54 is employed for black-and-white threshold processing of the metallographic images, where pixels representing fibers and resin are replaced with “255” and “0”, respectively. The fiber volume fraction is then determined by calculating the percentage of pixels representing fibers. With adjustments to different thresholds, it can also be used to calculate the porosity of composite materials. It is important to note that the imaging method has high requirements for polishing precision of the samples, and the fibers must be polished completely. Otherwise, the identification effectiveness may be significantly compromised. Additionally, the limited characterization zone, uneven dispersion of fibers caused by processing, and random distribution of pores may lead to significant errors in volume fraction calculations in different selected regions.

#### 2.2.2. Porosity

In addition to obtaining the material porosity through metallographic image processing, it can also be measured using the density method. Specifically, considering the known fiber volume fraction *V_f_*, the theoretical density *ρ*_0_ is calculated assuming that the composite material is without voids. The actual density *ρ* is then measured using the Archimedes buoyancy method, and the porosity φ is calculated according to Equations (4) and (5).

(4) $$\rho_{0}=V_{f}\rho_{f}+\left(1-V_{f}\right)\rho_{r}\;$$

(5) $$\varphi =\frac{V-V_{0}}{V}\times 100\%=\frac{\rho_{0}-\rho }{\rho_{0}}\times 100\%\;$$

where *ρ*_0_ is the theoretical density of the void-free composite specimen, *ρ* is the actual density of the specimen, *V* is the actual volume of the specimen, and *V*_0_ is the theoretical volume of the specimen.

The porosity of composite panels and CF/PA6 window frames is inspected by ultrasonic C-scan technology. The detection is performed using the bottom reflection method. In the case of a uniform and defect-free material, the bottom reflection wave exhibits minimal attenuation and a larger amplitude. Conversely, when defects are present in the material, the bottom reflection wave experiences significant attenuation and a smaller amplitude.

#### 2.2.3. Mechanical Property

Mechanical property tests include two parts: CF/PA6 composite materials and CF/PA6 winding compression-molded window frames.

Mechanical property test samples of CF/PA6 composite materials were prepared using the same compression molding process as the window frames to evaluate the influence of molding process parameters on the quality of composite material compression molding. Additionally, the testing results were used to obtain the engineering mechanical parameters (Table 1) required for simulation calculations of CF/PA6 composite materials and chopped CF/epoxy composite materials. The laying, cutting, and testing processes of the relevant samples followed ASTM standards, as detailed below:

Regarding the 0° tensile and 90° tensile tests, refer to ASTM D3039 [26], where the dimensions of the 0° tensile specimen are 250 mm (length) × 15 mm (width) × 1 mm (thickness), with reinforcing patches of 55 mm length attached at both ends and a testing length of 140 mm retained in the middle. The dimensions of the 90° tensile specimen are 175 mm (length) × 25 mm (width) × 2 mm (thickness), without the need for reinforcing patches. The testing speed is set at 2 mm/min, with strain gauges attached to the middle part of the specimen during testing to collect strain data, and a strain range of 1000–3000 με is taken to calculate the modulus of the specimen. Each test condition is repeated five times, and the experimental results are averaged.

Regarding the 0° compression test, refer to ASTM D6641 [27], with compression dimensions of 140 mm (length) × 12 mm (width) × 2 mm (thickness), with reinforcing patches of 63 mm length attached at both ends and a testing length of 14 mm retained in the middle. The strain collection and modulus calculation methods are the same as those for tensile testing.

Regarding the 0° bending test, refer to ASTM D7264 [28], using a three-point loading method. The specimen dimensions are 150 mm (length) × 13 mm (width) × 4 mm (thickness). The span-to-thickness ratio of the specimen is 32:1, with the two lower supports spaced 128 mm apart. The testing speed is set at 1 mm/min, and the test ends when the specimen fractures into two halves or when the stress decreases to 40% of the maximum stress value. The strain collection and modulus calculation methods are the same as those for tensile testing.

Regarding the interlaminar shear strength test (i.e., short beam shear test), refer to ASTM D2344 [29], using a three-point loading method. The specimen dimensions are length: width: thickness = 6:2:1, with a cutting size of 18 mm (length) × 6 mm (width) × 3 mm (thickness). During testing, the span-to-thickness ratio of the two lower supports is 4:1, with a span of 12 mm. The testing speed is set at 1 mm/min, and the test ends when the specimen fractures, the force decreases to 30%, or if there is no obvious stress drop. The test is manually stopped when the upper pressing head displacement exceeds the nominal thickness of the specimen or when the stress rises to 3000 N, to prevent excessive force from squeezing the specimen and damaging the supporting fixture. The strain collection and modulus calculation methods are the same as those for tensile testing.

Regarding the ±45° in-plane shear test, refer to ASTM D3518 [30]; the specimen layup sequence is [45/−45]_4_. The specimen dimensions are 250 mm (length) × 25 mm (width) × 5 mm (thickness), without the need for reinforcing patches. The tensile speed, strain collection, and modulus calculation methods are the same as those for tensile testing.

**Figure 4:**
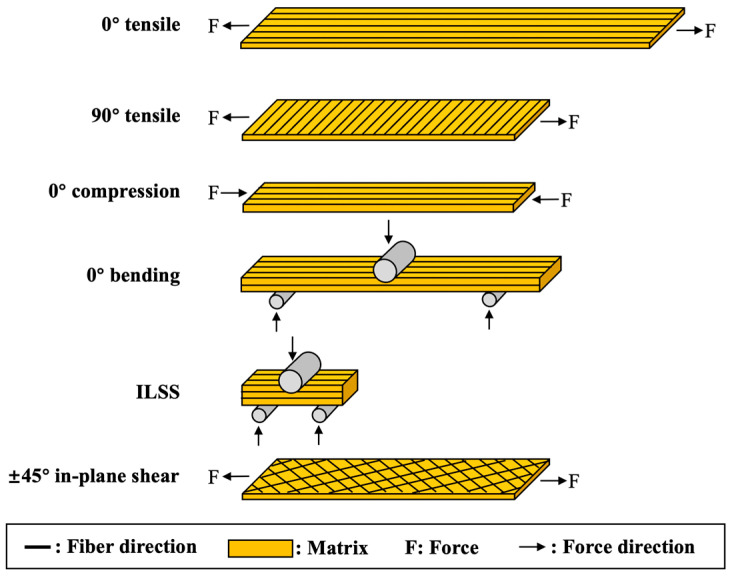
Schematic for the mechanical properties tests of CF/PA6 composite materials.

The mechanical property tests for CF/PA6 composite materials are schematically shown in Figure 4.

**Figure 5:**
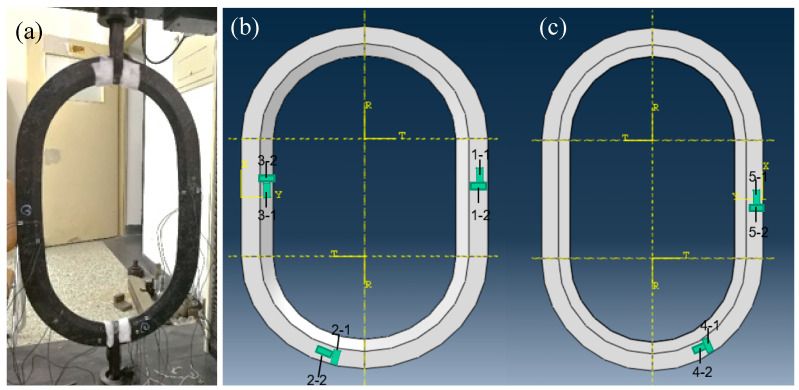
(**a**) The mechanical performance testing of window frame samples and (**b**,**c**) the strain data acquisition positions on the front and back.

In the mechanical performance testing experiments of the window frames, a simplified test method was used to evaluate the longitudinal tensile properties of two composite material window frames: the continuous CF/PA6 winding compression molded window frame and Quantum Company’s Lytex-4149 chopped CF/epoxy compression-molded window frame. As shown in Figure 5a, to prevent damage to the window frame samples by the fixtures, the top sections of the aircraft window frames were wrapped with thick, soft fabric longitudinally, and flexible fiber bundles were used for wrapping and subsequent load application. Before testing, five points were selected for strain gauge placement on the inner and outer sides of the middle section, as well as on the front and back corner sections of the window frames, and strain data were collected along both the hoop and radial directions (Figure 5b,c), to collect strain data in different directions at different points during the tensile test. The testing speed was set at 2 mm/min. The experimental stop condition was set to a deformation of 1% or initial fracture of the specimen.

### 2.3. Simulation

#### 2.3.1. Material Properties

In order to comprehensively evaluate the mechanical performance of the window frames prepared by the novel winding compression molding process proposed in this paper, further finite element analyses (FEA) were conducted based on the experimental tests mentioned above. Additionally, a comparative evaluation of the performance of window frames produced using three different materials—a continuous CF/PA6 thermoplastic composite winding compression molding frame, a chopped CF/epoxy thermosetting composite compression molding frame, and a 6061-T6 aluminum alloy frame—was further performed. Three groups of material properties used in the FE model are presented in Table 1.

#### 2.3.2. Finite Element Models

**Figure 6:**
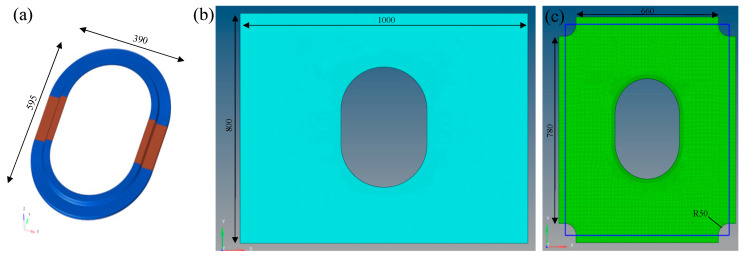
Finite element models: (**a**) window frame, (**b**) skin for tensile, (**c**) skin for shear. (Unit: mm).

As shown in Figure 6, the models used in the finite element analysis section not only establish the model of the window frame itself but also incorporate additional loading skin parts to simulate the actual load transfer scenario from the aircraft fuselage to the window frame. Specifically, the window frame is modeled using CHEXA solid elements, with CF/PA6 and CF/epoxy anisotropic materials using the MAT9 property card, aluminum alloy isotropic materials using the MAT1 property card, and the element property defined by the PSOLID card. The material coordinate system for the curved area of the window frame adopts a cylindrical coordinate system, while the remaining straight areas adopt a Cartesian coordinate system. The skin is made of unidirectional T800 carbon fiber/epoxy prepreg, with a layup pattern of [45/−45/45/−45/0/90/0/90/0/−45/45/−45/45]. Material properties are listed in Table 2. Modeling is done using CQUAD4 shell elements, with material cards utilizing the MAT8 card, and properties defined by the PCOMP card. The connection between the skin and the window frame is modeled using GLUE contact type to simulate the bonding between the window frame and the skin.

#### 2.3.3. Loading Scheme and Boundary Conditions

**Figure 7:**
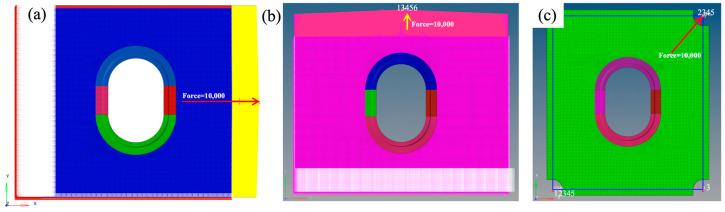
Finite element model loading scheme and boundary conditions: (**a**) X-directional tensile scheme, (**b**) Y-directional tensile scheme, and (**c**) diagonal shear scheme.

The window frame structure serves as an open structure on the aircraft sidewall and primarily experiences tensile and shear loads induced by fuselage twisting during flight. Therefore, three loading schemes (Figure 7) were adopted in this study to assess the mechanical performance of the window frame: the X-directional tensile scheme, which applies tensile loading along the longitudinal axis of the fuselage; the Y-directional tensile scheme, which applies tensile loading along the lateral axis of the fuselage; and a diagonal shear scheme simulated by pure shear loading through fixture formation. In both tensile loading schemes, all degrees of freedom in the restrained fixture segment area were constrained, while the degrees of freedom in direction 23,456 were constrained in the loaded segment, along with the freedom of direction 3 on both sides. Loading was applied using RBE3 elements, with a +10,000 N load in the X direction for X-directional tensile analysis and a +10,000 N load in the Y direction for Y-directional tensile analysis. In the shear loading mode, the fixture clamping effect was simulated using one-dimensional BAR elements. The degrees of freedom in direction 12,345 were constrained at the clamping angle, and the degrees of freedom in the load direction and rotation direction were constrained at the loading angle. Additionally, the degrees of freedom outside the surface were restrained at the other two corners to simulate hinges. A +10,000 N load was applied in the loading segment. The maximum strain ε_max_ was outputted and compared with the experimental true failure strain ε_ult_ to evaluate the load-bearing capacity of the material.

## 3. Results and Discussion

### 3.1. Performance of CF/PA6 Filaments

**Figure 8:**
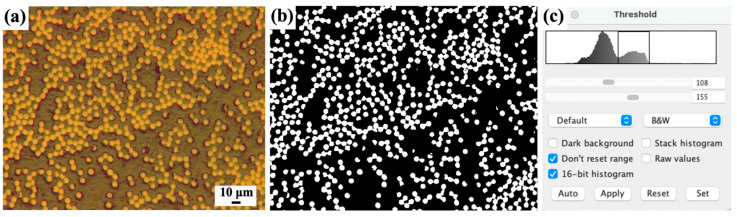
Image threshold method used for measuring the fiber content of CF/PA6 composites: (**a**) typical OM image of the CF/PA6 composite cross-section after polishing, (**b**) the black-and-white image of (**a**) processed by ImageJ threshold controlling, and (**c**) the software interface screenshot of ImageJ used for calculating the fiber volume fraction.

The properties of continuous CF/PA6 filaments used for preform winding of the window frame are summarized in Table 3 and Table 4 through detailed physical and mechanical property tests. It can be observed that the fiber volume fraction measured by the metallographic imaging method (Figure 8) is in good agreement with the theoretical value, reaching 42 vol %. The porosity of the filaments is 6.38%, with a density of 1.32 g/cm^3^, which is lower than the theoretical density of 1.41 g/cm^3^. This may be attributed to insufficient impregnation of PA6 on CF during the impregnation process through the impregnation cross-head. Additionally, the effective compaction distance at the exit of the filament shaping die also affects the porosity. In terms of mechanical properties, continuous CF/PA6 material exhibits typical anisotropic characteristics. It demonstrates superior 0° tensile strength and modulus compared to Quantum Lytex-4149 CF/epoxy and 6061-T6 aluminum alloy, but presents a significant shortfall in the 90° direction. This is the fundamental reason for the unique performance characteristics of the window frame to be introduced later.

### 3.2. Optimization of Winding Compression Process

#### 3.2.1. Process Parameter Optimization

**Figure 9:**
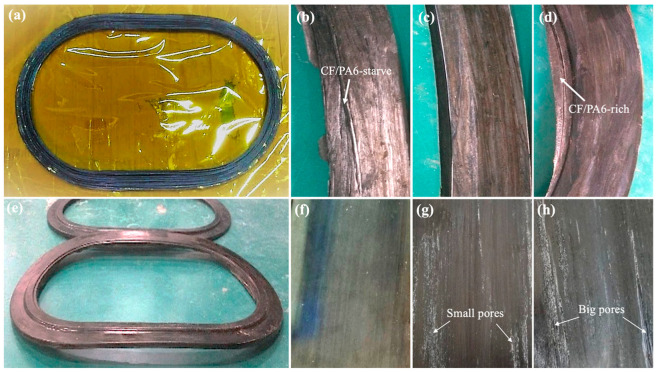
(**a**) The winding CF/PA6 window frame preform prepared under 13 N pretension force. Effects of the preform mass on the quality of compression-molded CF/PA6 frame: (**b**) 600 g, (**c**) 700 g, (**d**) 800 g. (**e**) Effects of the compression dwell time on warpage of the CF/PA6 composites: when the time is less than 300 s, warping deformation is prone to occur due to the release of residual stress. Effects of the mold temperature on surface quality of the CF/PA6 composites: (**f**) 160 °C with smooth surface, (**g**) 180 °C with small pores, (**h**) 200 °C with many big pores.

The main controlled process parameters during winding compression molding are winding tension, preform mass, mold temperature, and holding time. The relevant optimization process and experimental results are shown in Figure 9. The winding tension mainly originates from the unwinding and pre-tensioning of CF/PA6 filaments. To avoid excessive relaxation between wound layers leading to high porosity, while also aiming to maintain a high winding speed to ensure high production efficiency, a winding tension of 13 N was selected to fabricate the window frame preforms. As shown in Figure 9a, noticeable roughness and porosity are observed on the surface of the wound window frame preform, but overall, the morphology is good and the structure is stable, meeting the requirements for subsequent compression molding. In fact, as will be discussed later regarding the results of the window frame compression molding, it is also coincidentally shown that the novel process of thermoplastic composite winding compression molding using the preform proposed in this paper has a high tolerance and good potential for industrial application.

After making the preform, when proceeding to the next step of molding, if the feed mass is too low, it can result in incomplete mold filling, leading to product defects such as insufficient resin. Conversely, excessive feed material can cause fiber overflow and deformation. According to the theoretical volume of the window frame mold and the theoretical density of the material, the theoretical mass of the wound preform is 690 g. However, considering the relatively high porosity of the preform during winding, we selected three preforms with different masses of 600 g, 700 g, and 800 g for comparison in molding quality. In addition, while increasing the mass, the preform must also be matched with the cavity of the compression mold without affecting closure. Therefore, we mainly adjusted the thickness of the winding mold to produce preforms of different masses. From the actual appearance results of the molded parts in Figure 9b–d, it can be seen that the window frame samples obtained from the 600 g preform exhibit noticeable resin starve at the edges and center surface. The 800 g molded product shows a relatively full and dense center surface, but noticeable composite material overflow occurs at the edges, indicating overfeeding of the preform. The 700 g sample exhibits smooth and defect-free surfaces and edges. However, to ensure efficient compactness and minimize porosity, the final preform mass was set to 720 g, representing a 4.3% overfeeding.

The impact of the molding die temperature on the surface quality of CF/PA6 is illustrated in Figure 9f–h. It can be observed that when the temperature exceeds 160 °C, numerous white bubbles appear on the surface of the composite material, indicating that the residual gas within the material cannot be effectively expelled or shrunk to a sufficiently small volume. At the same time, the active movement of PA6 polymers prevents rapid cooling and stabilization, leading to crystallization and significant volume reduction, causing material shrinkage and potentially severe shrinkage defects. Therefore, we selected 160 °C as the final molding temperature for CF/PA6 composite material window frames.

The holding time has a significant influence on warpage deformation (Figure 9e). This is because the molding stage primarily involves cooling the PA6 polymer from its melting temperature (260 °C) to the mold temperature (160 °C). This process consists of two stages: (1) the stage of high-temperature resin flow and impregnation, during which the main purpose of holding pressure is to ensure uniform flow and redistribution of PA6 resin within the preform, accompanied by bubble elimination; (2) the stage of low-temperature solidification, where amorphous PA6 undergoes cold consolidation and rearrangement of crystalline structure. A longer holding time results in a smaller temperature gradient in the thickness direction and more complete crystallization of the material. Thus, a longer holding time is conducive to reducing residual stresses within the specimen, and the resulting window frame structure is more stable after demolding. However, considering production efficiency, we chose 300 s as the final holding time. Achieving faster production rates can be addressed by implementing a separate post annealing and shaping fixture.

**Figure 10:**
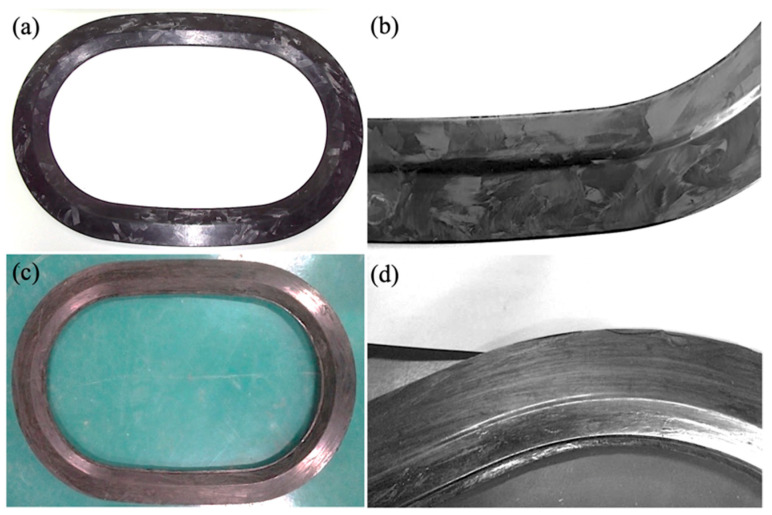
Window frame manufactured from (**a**,**b**) chopped CF/epoxy thermosetting composites via compression molding, and (**c**,**d**) continuous CF/PA6 thermoplastic composites via winding compression molding.

Ultimately, the CF/PA6 winding compression window frames are prepared using the critical parameter combination of a winding tension of 13 N, preform overfill mass of 4.3%, molding temperature of 160 °C, and holding time of 300 s. The obtained CF/PA6 frame, along with the comparison with the short-cut CF/epoxy window frame, are shown in Figure 10. It can be seen that they both exhibit good appearances.

It is worth noting that the technique employed in this study achieves the manufacturing of rotary structures with high-quality by using only simple and low-cost winding equipment with the assistance of post-compression molding. However, in future improvement studies or industrial batch production, the use of high-precision automation control systems or robots [31,32,33] for preform winding molding remains recommended to enhance the accuracy of fiber laying paths and the production stability of window frames through this novel winding compression technology.

#### 3.2.2. Porosity

The porosity of composite materials has a direct impact on their mechanical properties and usability. Bascom and Romans [34] observed that reducing the void content from 5% to less than 0.1%, leads to a 50% increase in ILSS. Stamopoulos [35] reported 11% reduction of ILSS for ~3% of porosity. The aerospace industry commonly uses 2% void volume fraction as the acceptable level of laminate porosity [36,37].

**Figure 11:**
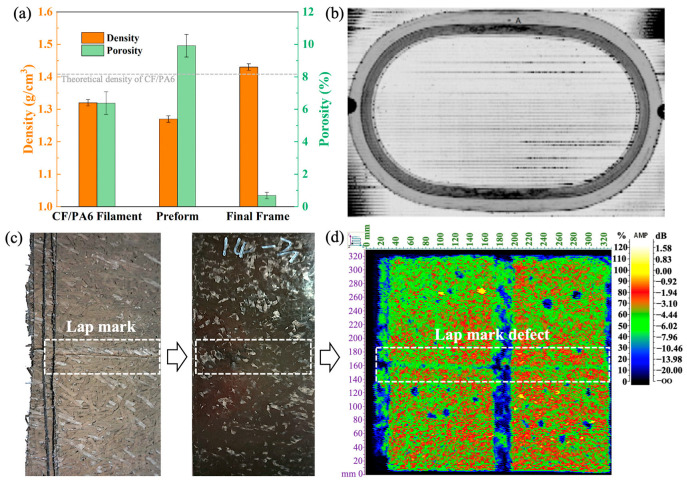
(**a**) The density and porosity variations of CF/PA6 composites at different forming stages; (**b**) ultrasonic C-scan of the CF/PA6 winding compression window frame; (**c**) Lytex-4149 chopped CF/epoxy thermosetting composite panel, and (**d**) ultrasonic scanning results of (**c**), where the lap marks are clearly visible. (Note: The porosity of the window frame is obtained through the metallographic imaging method, and, due to the actual density of the window frame exceeding the theoretical density, the Archimedes density method is no longer applicable).

The actual density and porosity of CF/PA6 composites at various forming stages are shown in Figure 11a. It can be observed that the porosity of the prepreg filament is 6.38% due to incomplete impregnation and compaction during filament shaping, while the porosity of the winding preform reaches 9.92%. However, after the final compression molding, the porosity decreases to only 0.69%, far below the 2% limit allowed for civil aviation composite structural components. Meanwhile, the density increases to 1.43 g/cm^3^, exceeding the theoretical density of 1.41 g/cm^3^ in Table 4. This is because, during winding, the high rapid winding speed and low pressure result in insufficient compactness of the preform and high porosity, leading to a decrease in density. After compression molding, under the combined action of higher pressure and the overflow gap between the molds, some PA6 resin flows out with the voids during compaction, resulting in a loss of resin matrix quality, indirectly increasing the fiber content, and consequently exceeding the theoretical density of the window frame. This also indicates that although the porosity of the preform is relatively high, the aircraft window frame samples prepared through the innovative winding compression combination method in this paper can still achieve excellent molding quality. Investigation through ultrasonic C-scan (Figure 11b) also demonstrates that the aircraft window frame samples prepared using the winding compression process are virtually free of void defects, further confirming the feasibility of the process. However, when performing ultrasonic C-scan on the chopped CF/epoxy specimens (Figure 11c,d), it is evident that discontinuous fibers caused by necessary cutting processes during material laying result in noticeable lap marks in the C-scan images. This inherent process flaw can significantly affect the performance and operational safety of the products.

### 3.3. Window Frame Mechanical Properties

This study conducted Y-directional tensile test experiments on the window frames and simultaneously established corresponding Abaqus finite element analysis (FEA) under the same loading conditions. By comparing experimental and simulation results, the reliability of the established FE model was firstly evidenced. Then, the established models were used to evaluate the X-directional tensile, Y-directional tensile, and diagonal shear properties of aircraft window frames manufactured from three different materials to simulate three typical loading scenarios.

#### 3.3.1. Tensile Experiments on Composite Window Frames

**Figure 12:**
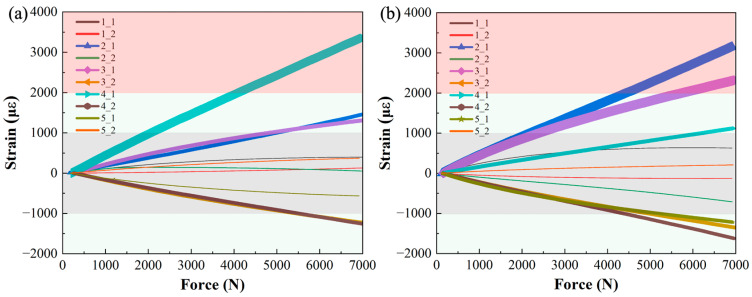
Tensile test strain curves of (**a**) CF/PA6 winding compression frame, (**b**) chopped CF/epoxy frame. (The strain is divided into three areas by absolute strain values of |με| < 1000, 1000 < |με| < 2000, and |με| > 2000).

**Figure 13:**
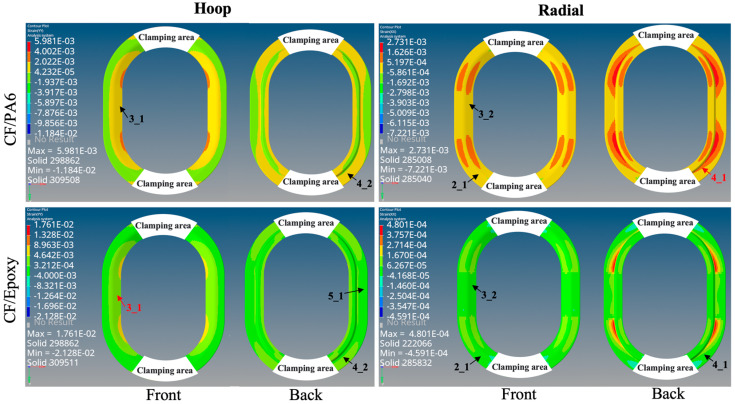
The strain maps of the CF/PA6 winding compression window frames and the chopped CF/epoxy compression molded window frames at a load of 7000 N.

The experimental test procedure on the Y-directional tensile properties of CF/PA6 winding compression window frames and chopped CF/epoxy compression-molded window frames are shown in Figure 4. In the actual experimental testing process, we simplified the testing method by not using peripheral loading skins. the maximum principal strain data at characteristic point locations was selected for evaluating the window frame performance. Under a 7000 N tensile load, the tested experimental strain data and the Abaqus strain contour plots are shown in Figure 12 and Figure 13, respectively. For clarity and analysis purposes, the strain data in Figure 11 are divided into three regions based on absolute strain values of |με| < 1000, 1000 < |με| < 2000, and |με| > 2000. The critical strain response locations are annotated in Figure 13, where red represents the principal strain.

It can be observed that they have similar strain distributions for CF/PA6 and CF/epoxy frames. For the CF/PA6 winding compression window frames, both experimental and simulated maximum principal strains occur at position 4_1, i.e., the radial direction at the maximal curvature corner. This is attributed to the occurrence of pure interlayer shear or 90° tensile stress due to the absence of fiber reinforcement in the radial direction, making it prone to delamination and fiber fracture and resulting in considerable deformation. The maximum experimental and simulation strains are 3347 με and 2731 με, respectively, showing good agreement. In contrast, for the chopped CF/epoxy window frames, the maximum strain occurs at position 3_1 (Note: although position 2_1 also exhibits strains of the same magnitude, it is more likely influenced by stress concentration in the loading area, which is considered less objectively valuable and thus not discussed here. The strain level at this point will be objectively analyzed in Section 3.3.2). This indicates that the maximum load is distributed along the straight sections on both sides of window frame, indicating that the window frame is primarily subjected to longitudinal tensile loads. The maximum strain in experimental measurements and simulation contour plots is 2317 με and 3316 με, respectively, also showing good correspondence. Due to the high-performance continuous fiber reinforcement in the circumferential direction of the CF/PA6 winding compression window frames, the strains at position 3_1 are experimentally measured at 1311 με and simulated at 1312 με, which are almost identical and significantly lower than the strain levels of the chopped CF/epoxy.

In summary, through the comparison of the above principal strain experimental data with simulations, good correspondence is achieved, demonstrating the reliability of the FE models. This provides a foundation for subsequent comprehensive analysis of window frame performance. It also preliminarily indicates that CF/PA6 winding compression window frames exhibit superior hoop performance but may be weaker radially, and under higher load levels, there may be a risk of preferential occurrence of shear delamination or fiber fracture failure at the maximum curvature.

#### 3.3.2. Performance Evaluation through Simulation Analysis

The lack of precise position correspondence between the experimental strain gauge and the simulated strain maps, and the simplified loading test method selected, may lead to stress concentration areas in the window frames, affecting the true distribution of loads. However, the accuracy of the window frame FE model is validated through the comparison of the aforementioned experimental and simulation results. Therefore, based on this, in this section, a more reasonable FE model is re-modeled for a more objective and accurate analysis of stress levels and positions in the window frames according to the standard window frame testing method [17]: (1) The skin of the simulated fuselage was added, and the window frames were indirectly loaded through this skin, which makes the simulation more representative of real conditions. (2) Three loading schemes are added: the X-directional tensile scheme and Y-directional tensile scheme along the longitudinal and lateral sides of the fuselage, respectively, and diagonal tensile loading with pure shear scheme through the fixture. (3) Three materials are compared: continuous CF/PA6 thermoplastic composite winding compression molding, chopped CF/epoxy thermosetting composite compression molding, and 6061-T6 aluminum alloy. By comparing with the real window frame, the performance characteristics of the CF/PA6 winding compression molding window frames in this paper are intended to be objectively evaluated.

**Figure 14:**
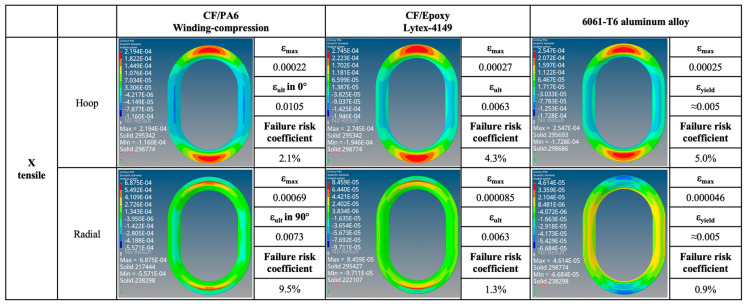
X-directional tensile strain map. (Note: Failure risk coefficient = ε_max_/ε_ult_ × 100%).

**Figure 15:**
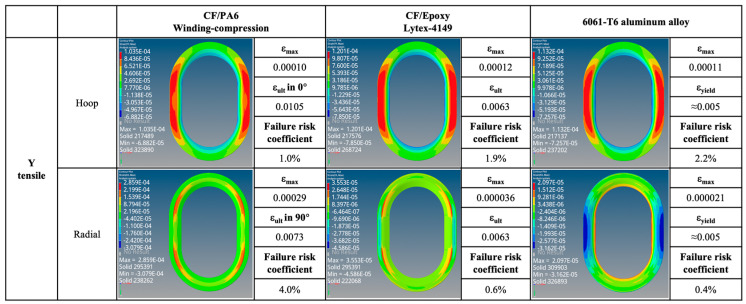
Y-directional tensile strain map. (Note: Failure risk coefficient = ε_max_/ε_ult_ × 100%).

**Figure 16:**
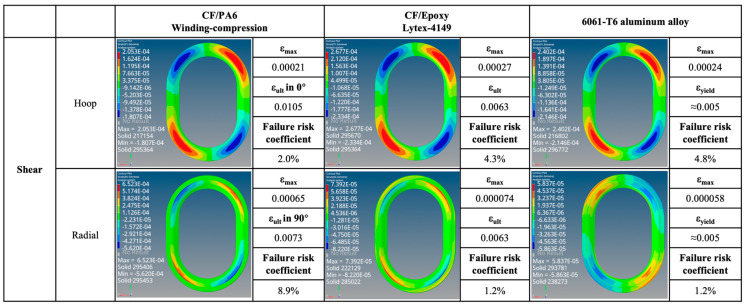
Shear strain map. (Note: Failure risk coefficient = ε_max_/ε_ult_ × 100%).

**Figure 17:**
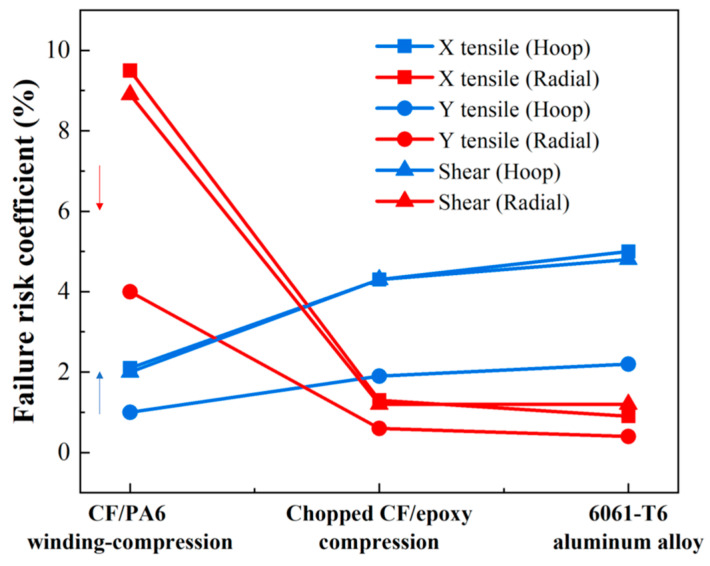
Performance summary and comparison of the window frames manufactured with three kinds of materials. Where the red arrow implies the properties can be adjusted downwards in the future, while the blue arrow indicates the properties should be improved.

The strain maps and failure probabilities of the three materials under three test loading schemes are summarized in Figure 14, Figure 15, Figure 16 and Figure 17. It can be seen that under the same loading scheme, the three materials exhibit similar experimental values and trends, while the chopped CF/epoxy and aluminum alloy frames show similar radial and hoop stress levels under all loading schemes. Specifically, CF/PA6 exhibits significant performance advantages over the other two materials in the hoop direction. Under a load of 10,000 N, it only reaches a level below 2% of the material intrinsic failure strain, which is about half those of the other two materials. This is due to the continuous fiber reinforcement effect. However, it shows significant deficiencies in the radial direction, reaching a level of 10% of the intrinsic failure strain, about ten times higher than the other two materials’ levels of around 1%. This is because in the radial direction, the CF/PA6 frame lacks fiber reinforcement and mainly relies on the resin matrix and CF/PA6 interface. As shown in Table 4, the strength in this direction, i.e., the 90° strength and interlayer shear strength, is only about 26–65 MPa, far lower than the 0° fiber direction strength of 1357 MPa and the other two materials’ strengths of 217 MPa (chopped CF/epoxy) and 303 MPa (6061-T6 aluminum alloy). Therefore, significant strains occur in the radial direction, representing a higher risk of failure. However, interestingly, during the actual experimental test process, when the load reached 7000 N, the Lytex-4149 thermosetting aircraft window frame emitted a crisp fracture sound, possibly corresponding to internal fiber damage, fracture, and delamination. In contrast, although the T700/PA6 thermoplastic aircraft window frame experienced significant strains in the corner radius direction, it did not exhibit significant damage or fracture until loaded to 9000 N, demonstrating a higher damage tolerance. This is attributed to the higher toughness of thermoplastic composites compared to thermosetting composites. Nonetheless, for higher safety, future design optimizations should focus on balancing the hoop–radial comprehensive performance, for example, by reducing the number of hoop fiber winding layers and compensating by increasing the radial fiber laying layers as indicated by the blue and red arrows in Figure 17.

Additionally, by comparing Figure 15 and the previous Figure 13, it can be observed that after applying skin loading to the window frames, the strain levels significantly decrease due to the uniform distribution of external loads onto the window frame. Under X-directional tension, the maximum principal strain in the hoop and radial directions of the window frame occurs at the apex of the arc. In Y-directional tension, the positions of maximum principal strain in the hoop direction for both CF/PA6 and CF/epoxy have shifted from the original inner side of the window frame (3_1) to the outer side (1_1), corresponding to the different applied methods of external loads. The radial direction still exhibits weaker stress concentrations at 2_1 (also 4_1). In shear testing, the maximum principal strain in both hoop and radial directions for all three materials occurs at position 2, with symmetrical angles displaying symmetrical strains but with positive and negative values. This once again emphasizes the importance of the mechanical properties at position 2 for the overall performance of the window frame.

## 4. Conclusions

This study proposes an innovative winding compression molding process for continuous production of aircraft window frames using self-prepared continuous carbon fiber-reinforced thermoplastic composite filaments. Through process parameter optimization, high molding efficiency, low porosity, and high-performance manufacturing of CF/PA6 window frames were achieved. Combined with experimental testing and finite element analysis, the performance of window frames made of three different materials was comparatively evaluated, leading to the following conclusions:

(1) The production cycle of the new winding compression process can be controlled within 5 min. Key process parameters include a winding preheating infrared heater temperature of 400 °C, a winding speed of 10 m/min, a winding pre-tension force of 13 N, and a preform feeding mass of 720 g. The preform is then preheated at 260 °C and stamping molded at 160 °C under a pressure of 4 MPa, with a holding time of 300 s, and additional post-molding shaping fixtures are applied after demolding.

(2) Winding molding ensures efficient preform manufacturing cycle for window frames, while molding further consolidates the preform during final shaping, ensuring low porosity and high performance. The density increased from 1.27 g/cm^3^ of the preform to 1.43 g/cm^3^, and the porosity decreased from 9.92% of the preform to 0.69%, meeting aviation application standards.

(3) In the hoop direction, the performance of CF/PA6 window frames with continuous fiber winding reinforcement is much higher than that of chopped fiber compression molding and aluminum alloy. Although the radial performance is weaker compared to the competitors, it still exhibits high crack propagation resistance and damage tolerance: when the load is applied up to 7000 N, the thermosetting aircraft window frame of Lytex-4149 emits a crisp cracking sound, while the toughness of the T700/PA6 thermoplastic aircraft window frame is much higher, and no obvious cracking occurs even when loaded up to 9000 N. The simulation analysis results indicate that the maximum strains occur respectively at the apex of the arc under X-directional tension, at the straight segment and corners under Y-directional tension, and at the corners under shear.

(4) This study provides an innovative process reference for high-speed continuous fiber-reinforced thermoplastic composite product molding, ranging from CF/PA6 to high-performance CF/polyetheretherketone (CF/PEEK). The limitation is that the current experimental approach relies solely on resin support in the radial direction, leaving significant room for performance improvement. Further attempts to achieve balanced hoop–radial performance by appropriately increasing transverse ply reinforced layers deserve future investigation, with the potential to achieve significant weight reduction objectives. During the winding preforming stage, high-precision automation control or robot winding methods should also be considered for enhancement in the future to increase accuracy and batch stability.

## Figures and Tables

**Table 1:** The material parameters of CF/PA6, CF/epoxy, and aluminum alloy used in this paper for the FEA on the window frame properties.

Materials	E_11_	E_22_	E_33_	γ_12_	γ_13_	γ_23_	G_12_	G_13_	G_23_	Density	Ultimate Failure Strain, ε_ult_
Units	GPa	GPa	GPa				GPa	GPa	GPa	g/cm^3^	
UD T700/PA6 (40 vol.%)	86	4.94	4.94	0.3	0.3	0.3	2.22	2.22	1.9	1.4	0.0105 (0°) 0.0073 (90°)
Chopped CF/epoxy Lytex-4149	34.5	34.5	8.6	0.3	0.3	0.3	0.82	0.82	0.33	1.48	0.0063
6061-T6 aluminum alloy	70			0.3						2.7	≈0.005 (yield strain) [25]

Note: for CF/PA6, E_11_ = 0° tensile modulus, E_22_ = E_33_ = 90° tensile modulus, G_12_ = G_13_ = ±45° tensile modulus, G_23_ = E_22_/[2 × (1 + γ_12_)], γ_1_ = γ_2_ = γ_3_ = 0.3: a general estimate value for fiber composites.

**Table 2:** The material parameters for the skin.

Materials	E_1_	E_2_	E_3_	γ_1_	γ_2_	γ_3_	G_12_	G_13_	G_23_	Ply Thickness
Units	GPa	GPa	GPa				GPa	GPa	GPa	mm
T800/Epoxy	141	8.54	8.54	0.3	0.3	0.45	4.43	4.43	3.1	0.184

**Table 3:** CF/PA6 filament physical properties.

No. of Specimen	1	2	3	4	5	6	Avg.
CF-T700SC (μm)	7	7	7	7	7	7	7
Monofilament fiber dosage	36 k	36 k	36 k	36 k	36 k	36 k	36k
CF/PA6 filament diameter (mm)	1.97	1.98	2.05	2.15	2.11	2.1	2.06
Fiber volume content (vol %)	46	45	42	39	40	40	42
Density (g/cm^3^)							1.32
Porosity (%)							6.38 (Density method determined)

**Table 4:** CF/PA6 filament mechanical properties.

Materials	T700/PA6	Quantum Lytex-4149 CF/Epoxy	6061-T6 Aluminum Alloy
Fiber-reinforced form	Continuous	Chopped	—
Fiber weight fraction (wt %)	50 (weight-loss method)	55	—
Theoretical density (g/cm^3^)	1.41	1.48	2.7
0° tensile strength (MPa)	1357	217	303
0° tensile modulus (GPa)	86	34.5	70
90° tensile strength (MPa)	26	217	303
90° tensile modulus (GPa)	4.9	34.5	70
0° compressive strength (MPa)	378	—	303
0° compressive modulus (GPa)	56	—	70
0° bending strength (MPa)	733	531	303
0° bending modulus (GPa)	80	31.7	70
Interlaminar shear strength (ILSS) (MPa)	65	44.8	—
±45° in-plane shear strength (MPa)	35	—	—
±45° in-plane shear modulus (GPa)	2.2	—	—

## Data Availability

Data are contained within the article.
